# Examining correlations between opioid dispensing and opioid-related hospitalizations in Canada, 2007–2016

**DOI:** 10.1186/s12913-020-05530-w

**Published:** 2020-07-22

**Authors:** Wayne Jones, Paul Kurdyak, Benedikt Fischer

**Affiliations:** 1 Centre for Applied Research in Mental Health and Addiction (CARMHA), Faculty of Health Sciences, Simon Fraser University, 515 W. Hastings St, Vancouver, British Columbia Canada; 2 Department of Psychiatry, University of Toronto, 250 College Street, 8th floor, Toronto, Ontario Canada; 3 Institute for Mental Health Policy Research, Centre for Addiction and Mental Health, 33 Russell Street, Toronto, Ontario Canada; 4 Institute for Clinical Evaluative Sciences (ICES), 2075 Bayview Avenue, Toronto, Ontario Canada; 5 Faculty of Medical and Health Sciences, University of Auckland, 85 Park Road, Grafton, Auckland, New Zealand; 6 Department of Psychiatry, Federal University of São Paulo (UNIFESP), R. Sena Madureira, 1500 - Vila Clementino, São Paulo, Brazil

**Keywords:** Canada, Dispensing, Hospitalizations, Morbidity, Opioids, Public health

## Abstract

**Background:**

High levels of opioid-related mortality, as well as morbidity, contribute to the excessive opioid-related disease burden in North America, induced by high availability of opioids. While correlations between opioid dispensing levels and mortality outcomes are well-established, fewer evidence exists on correlations with morbidity (e.g., hospitalizations).

**Methods:**

We examined possible overtime correlations between medical opioid dispensing and opioid-related hospitalizations in Canada, by province, 2007–2016. For dispensing, we examined annual volumes of medical opioid dispensing derived from a representative, stratified sample of retail pharmacies across Canada. Raw dispensing information for ‘strong opioids’ was converted into Defined Daily Doses per 1000 population per day (DDD/1000/day). Opioid-related hospitalization rates referred to opioid poisoning-related admissions by province, for fiscal years 2007–08 to 2016–17, drawn from the national Hospital Morbidity Database. We assessed possible correlations between opioid dispensing and hospitalizations by province using the Pearson product moment correlation; correlation values (r) and confidence intervals were reported.

**Results:**

Significant correlations for overtime correlations between population-levels of opioid dispensing and opioid-related hospitalizations were observed for three provinces: Quebec (*r* = 0.87, CI: 0.49–0.97; *p* = 0.002); New Brunswick (*r* = 0.85;CI: 0.43–0.97; *p* = 0.004) and Nova Scotia (*r* = 0.78; CI:0.25–0.95; *p* = 0.012), with an additional province, Saskatchewan, (*r* = 0.073; CI:-0.07–0.91;*p* = 0.073) featuring borderline significance.

**Conclusions:**

The correlations observed further add to evidence on opioid dispensing levels as a systemic driver of population-level harms. Notably, correlations were not identified principally in provinces with reported high contribution levels (> 50%) of illicit opioids to mortality, which are not captured by dispensing data and so may have distorted or concealed potential correlation effects due to contamination.

## Introduction

North America – Canada and the US - has been home to a severe public health crisis of opioid-related (i.e., morbidity and mortality) harms since the early 2000s, initially evolving in contexts of persistently high prescription opioid (PO) availability [1–3]. This burden, specifically, has involved excessively high levels of (e.g., overdose) mortality, even negatively affecting general population-based life expectancy in both jurisdictions [4, 5]. Concretely, there were 47,600 opioid-related deaths in the US, and 4100 in Canada, respectively, in 2017, representing similar population-based mortality rates [6, 7].

Most attention regarding acute opioid-related harms has focused on mortality outcomes, as recently further accelerated by widespread proliferation of highly toxic illicit opioid products [6, 8, 9]. Less attention has been given to morbidity outcomes, for example (non-fatal) opioid-related poisonings or disorders. Recent US data have demonstrated rising trends for opioid-related hospitalizations post-2000 [10–12]. Opioid-related hospitalizations among public drug plan beneficiaries in Ontario (Canada) increased by 55% from 2003 to 2013 [13].

North American studies have examined associations between population-levels of opioid-dispensing and adverse outcomes towards improved understanding of the systemic drivers of opioid-related harms. While substantive correlations between opioid-dispensing and mortality have been found for several populations and/or jurisdictions [14–16], select investigations have established similar correlations for morbidity outcomes. For example, strong associations were found between US-based national prescription volumes of major opioid formulations and related emergency department (ED) visits over a 10-year period (1995–2004 [17];). Moderai et al. established similar correlations for North Carolina (2008–2010); in Florida, associations between PO availability and morbidity were less pronounced than for mortality (2009 [18, 19];). Strong correlations have been ascertained between opioid-dispensing and opioid-related substance treatment admissions in Ontario (2005–2011 [14];). Furthermore, substantial congruence between opioid dispensing and related hospitalizations have been observed for Australian jurisdictions [20, 21].

In Canada, despite overall distinctly high levels of opioid availability, opioid dispensing has considerably varied inter-provincially, with up to 3-fold differences in standardized volumes. Over-time, an inversion trend has been observed: While levels of opioid-dispensing consistently increased until about 2012, some – but not all – provinces have featured substantial declines thereafter [22].

In the context of the acute yet differentiated developments regarding opioid availability and related harms, we examine possible over-time correlations between opioid dispensing and opioid-related hospitalizations across Canada for the period 2007–2016.

## Methods

We used national data on community-based PO dispensing across Canada – i.e., here the 10 provinces – for the study period; the vast majority (e.g., ~ 80%) of opioid medications are dispensed through retail pharmacies [22]. Raw prescription opioid dispensing data, including both branded and generic medications, were obtained from IQVIA’s Compuscript prescription database, drawn from a representative, stratified, and continuously refreshed sample of ~ 6000 retail pharmacies towards estimating pan-Canadian dispensing total through patented geo-spatial projection methodology as previously used in related analyses [23–25]. Raw aggregate PO dispensing data included the numbers of units dispensed by product name, formulation and strength. These data were converted to annual values of Defined Daily Doses (DDD) per 1000 population per day (DDD/1000/day) as the measuring unit, based on the WHO’s Anatomical Chemical Classification as well as population statistics, by province and year for the study period (2007–2016 [26, 27];. DDDs are the assumed average maintenance dose per day for a drug used for its main indication for an average adult. While limited in accuracy, DDDs are a widely utilized measurement unit for comparative population-level analyses, and superior to crude indicators like prescription numbers [28]. We restricted our data to ‘strong’ opioids, based on the WHO’s ‘pain ladder’ i.e. excluding ‘weak’ opioids (e.g., codeine), as well as methadone due to differential dispensing modes as well as limited involvement in hospitalizations [22, 29].

Data on opioid-related hospitalizations in Canada came from published data from the Canadian Institutes of Health Information (CIHI)‘s Hospital Morbidity Database (HMDB). The HMDB is a national data-holding on administrative, clinical and demographic information on inpatient separations from acute care hospitals (but excluding EDs) from provincial data-sources across Canada [30, 31]. Opioid-related hospitalizations were identified based on admissions and length-of-time in hospital considered related to ‘significant opioid poisonings’, with discharge abstracts as the primary diagnosis, or comprised selected secondary diagnostic types based on ICD-10-CA codes (T40.0–4 and T40.6). Respective data, reported in population-based rates (per 100,000) were available by province for fiscal years (i.e., defined formally in Canada from 01 April to 31 March of the correspondingly following year) 2007/2008–2014/2015 and 2016/2017 (i.e., excluding data for 2015/16, as these were not available from the original data-source).

Possible correlations between annual provincial opioid dispensing and opioid-related hospitalization rates were assessed through the respective nine over-time data-pairs of indicators for each of the provinces, matching annual opioid-dispensing with corresponding hospitalization data for the corresponding annual time units (e.g., 2016 and 2016/17). Using the Pearson product moment correlation, ten province-specific correlation values and their 95% confidence intervals (95%CI) were calculated using the cor.test function of the R stats package (2018) [32].

## Results

**Fig. 1:**
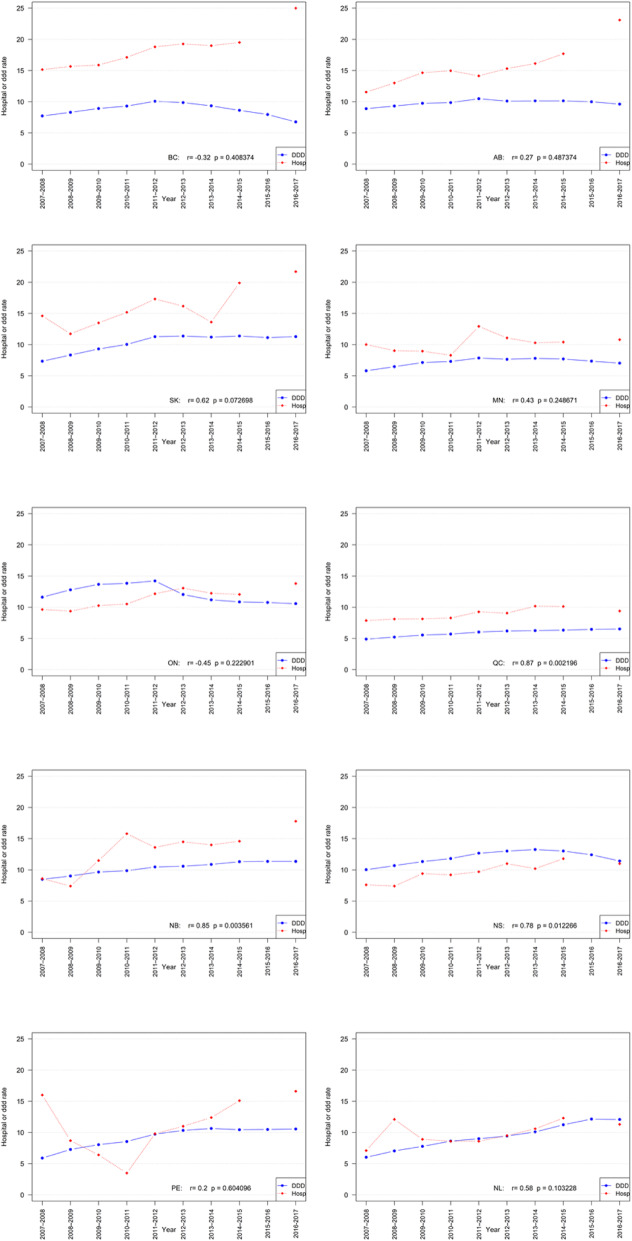
Rates and correlations of opioid dispensing and opioid-related hospitalizations, by province, in Canada 2007–2016 (visual display)

(see Table 1 for numeric data and Fig. 1 for corresponding visualizations).

**Table 1:**
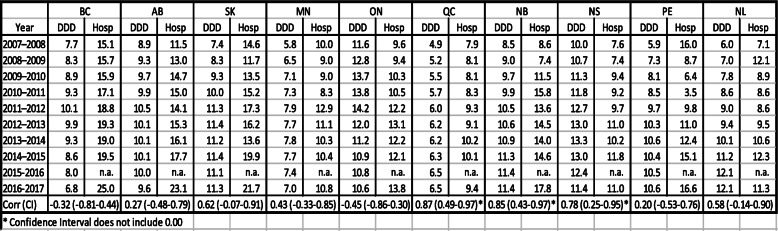
Rates and correlations of opioid dispensing and opioid-related hospitalizations, by province, in Canada 2007–2016

Legend: Acronyms of Provinces: *BC* British Columbia; *AB* Alberta; *SK* Saskatchewan; *MN* Manitoba; *ON* Ontario; *QC* Quebec; *NB* New Brunswick; *NS* Nova Scotia; *PE* Prince Edward Island; *NL* Newfoundland & Labrador

*DDD* Defined Daily Doses/1000population/day

*Hosp* Hospitalization

n.a.: data not available

For strong opioid dispensing, in 2007 (first observation year) the highest annual provincial rate was observed in Ontario (11.6 DDD/1000/day) and the lowest rate was in Quebec (4.9 DDD/1000/day) – constituting a > 2.3/1 ratio. Correspondingly, in 2016 (last observation year) Nova Scotia and New Brunswick featured the highest (11.4 DDD/1000/day) and Quebec had the lowest (6.5 DDD/1000/day) strong opioid dispensing rate – constituting a 1.7/1 ratio. All provinces featured higher strong opioid dispensing rates between the first and last observation year. While about half the provinces showed inverting trends in strong opioid dispensing – with annual levels initially rising and then decreasing – the other half featured consistent increases in strong opioid dispensing during the study period.

For opioid-related hospitalizations, in 2007/08 (first observation year) the highest annual rate was observed in Prince Edward Island (16.0/100,000) and the lowest rate was observed in Newfoundland and Labrador (7.1/100,000) – constituting a > 2.25/1 ratio. Correspondingly, in 2016/17 (last observation year) British Columbia featured the highest (25.0/100,000) and Quebec had the lowest (9.4/100,000) hospitalization rate – constituting a > 2.6/1 ratio. All provinces reported higher opioid-related hospitalization rates in 2016/17 compared to 2007/08. The majority of provinces featured varying (e.g., non-linear) trends in opioid-related hospitalization rates during the observation period.

The strongest correlation between opioid dispensing and hospitalization was observed for Quebec (*r* = 0.87, 95% Confidence Interval (95%CI): 0.49–0.97; *p* = 0.002); the weakest for Prince Edward Island (*r* = 0.20; 95%CI: − 0.53–0.76; n.s.). In a total of three provinces, significant correlations were observed: Quebec (see above); New Brunswick (*r* = 0.85; 95%CI: 0.43–0.97; *p* = 0.004) and Nova Scotia (*r* = 0.78; 95%CI: 0.25–0.95; *p* = 0.012); one province – Saskatchewan – (*r* = 0.073; 95%CI: − 0.07–0.91; *p* = 0.073) featured borderline significance (see Table 1 for data).

## Discussion

Opioid-related morbidity substantially contributes to the extensive opioid-related disease and economic burden observed across North America [33–35]. Strong increases in opioid-related hospitalizations post-2000 have been observed in the US, as well as other countries with rising opioid availability [10, 12, 21, 36, 37].

We examined possible correlations between opioid dispensing and opioid-related hospitalization rates across Canada in contexts of most provinces featuring inversion patterns for opioid dispensing during the study period (2007–2016 [22];). These patterns reflect recent reductions in opioid availability following a variety of restrictive measures (e.g., intensified prescription monitoring, de-scheduling of select opioid formulations, restrictive opioid prescription guidelines) in a wider context of continuously rising opioid-related mortality and morbidity outcomes in Canada [3, 38, 39].

We identified significant correlations between opioid dispensing and opioid-related hospitalization rates for three (QC, NB, NS) of the ten provinces, with borderline significance in a fourth (SK). These results, partially, add to an extensive body of evidence identifying correlations between the volume of opioid availability and health harm outcomes on population levels [14, 15, 19, 40]. While these correlations were identified in select provinces, they were not identified in most others. These selective findings, however may not be coincidental when considering key ecological dynamics. Importantly, opioid-related hospitalizations data may comprise incidents related to both licit and illicit opioids, as the data used do not discriminate between opioids by legal status [8, 10, 41]. However, opioid availability data are limited to levels of medical opioid dispensing only. These circumstances, consequently, may involve a ‘contamination’ of hospitalization data through illicit opioid-related cases and a subsequent distortion or concealment of possible correlation effects.

In Canada, population-levels of illicit opioid use have been relatively limited, compared with high levels of (medical and non-medical) PO use estimated at > 20% and > 5%, respectively, in peak years around 2010 [42]. However, there have been strong increases in the availability of – highly potent and toxic – illicit synthetic opioid products (e.g., fentanyl, carfentanyl products) in Canada in more recent years, linked to substantial increases in fatal poisonings due to acutely elevated risk properties of these substances [4, 43, 44]. In this context, we note that significant (including borderline) correlations between opioid dispensing and hospitalizations were found specifically in four of the five Canadian provinces (PEI, NS, QC, NB, SK) reporting the lowest levels of contributions (< 25%) of (mostly illicit) fentanyl or fentanyl-analogue product involvement among opioid-related mortality in 2017 (i.e., ‘low contamination’ provinces); conversely, no correlations were observed in the provinces (MN, ON, AB, BC) with fentanyl products identified as a contributor to mortality in the majority (> 50%) of fatalities in 2017 (i.e., ‘high contamination’ provinces [7, 8, 41];). Similarly differential – but consistent with our results – patterns of illicit opioid involvement in opioid-related hospitalizations have been shown in analyses for select individual provinces [45]. These – rather consistent – differentiation patterns in our ecological study results allow to plausibly speculate that, in the absence of illicit opioid-related ‘contamination’ effects, the strength of province-based correlation signals between opioid dispensing and hospitalizations likely would have been more pronounced.

In this context, our findings of select Canada-based correlations between opioid dispensing and opioid-related hospitalizations provide additional evidence on associations between population-level opioid availability and key adverse health outcomes [34, 46–48]. This association – at least while supply for non-medical opioid use mostly involved prescription opioid products – had simple but essential implications: The higher the volume of opioids dispensed into the population, the higher the levels of consequential morbidity or mortality harms [15, 49, 50]. This insight provided a crucial knowledge base for guiding opioid policy and medical practice control (e.g., prescription guidance) in the distinct contexts of traditionally high opioid availability in North America, combined with evidence of only limited effectiveness of opioids for chronic pain therapy [51–53]. Beyond these current contexts, where illicitly produced opioids have increasingly replaced pharmaceutical opioids for non-medical use, these insights may be helpful for settings where opioid availability is still low and preventive restraints can facilitate appropriate balance between opioid availability and related adverse health outcomes [5, 38, 54, 55]. As a potentially relevant limitation for the present analyses, we note that the (general population-level) data examined did not include additional patient-level descriptors, or other data variables that could have been used to examine possibly confounding factors in the correlations assessed.

## Conclusions

Our study identified correlations between over-time levels of prescription opioid dispensing and opioid-related hospitalizations in several provinces across Canada. These data further contribute to the evidence-base of population levels of prescription opioid availability and exposure, and opioid-related harms (e.g., morbidity, mortality). Correlations were mostly not observed in provincial jurisdictions where non-medical or illicitly produced opioids (e.g., fentanyls) have played an increasingly prevalent role in non-medical opioid use and harms in recent years, likely as an adverse consequence of recent restrictions on medical opioid availability (which may mask further correlations in regards to the specific data under study). Our study’s findings emphasize the need to reasonably limit the overall availability of and exposure to opioids in the population for general prevention.

## Data Availability

The datasets generated and/or analysed during the current study are in the public realm and/or are available from the authors on reasonable request.

## Abbreviations

PO: Prescription opioid
ED: Emergency department
DDD: Defined daily doses
